# Compartmental analysis: a new approach to estimate protein breakdown and meal response in health and critical illness

**DOI:** 10.3389/fnut.2024.1388969

**Published:** 2024-05-09

**Authors:** Nicolaas E. P. Deutz, Mariëlle P. K. J. Engelen

**Affiliations:** Center for Translational Research in Aging & Longevity, Texas A&M University, College Station, TX, United States

**Keywords:** amino acids, critically ill, ICU, stable isotopes, nutrition

## Abstract

**Purpose of review:**

This study aimed to discuss the use of the pulse stable isotope tracer approach to study changes in metabolism in healthy individuals and critically ill patients.

**Recent findings and conclusion:**

We found that in the postabsorptive state and healthy condition, intracellular protein breakdown and net intracellular protein breakdown, when calculated using the pulse tracer approach, are about double what has previously been reported using the more traditional primed-constant and continuous stable isotope approaches (600 versus 300 grams of protein/day). In critically ill patients, protein breakdown is even higher and calculated to be approximately 900 grams of protein/day, using the pulse tracer approach. Based on these data, we hypothesize that reducing protein breakdown in the postabsorptive state is key when trying to improve the condition of critically ill patients. Moreover, we also used the pulse tracer approach during feeding to better estimate the intracellular metabolic response to feeding. Our first observation is that endogenous protein breakdown does not seem to be reduced during feeding. We also have shown that when consuming a meal with a certain amount of protein, the biological value of that protein meal can be calculated with the pulse tracer approach. In conclusion, using the pulse stable isotope tracer approach to study protein kinetics in the postabsorptive state and during feeding expands our understanding of how dietary proteins can affect human protein metabolism. The intracellular protein synthesis stimulatory effect of a meal is an important factor to consider when calculating the exact protein requirements and needs, particularly in critical illness.

## Introduction

Meeting the enhanced needs of critically ill patients through optimal protein intake has been studied for many years. The current protein intake requirements (1) are based on the requirements established for healthy humans, to which a multiplication factor is added. However, protein turnover is highly upregulated in critically ill patients (2–4), suggesting an increased availability of amino acids as more amino acids are released intracellularly and into the circulation from protein breakdown (PB). Therefore, a higher disposal of those amino acids will become available for protein synthesis and other disposal routes. In steady-state conditions, the production of amino acids released from PB is in balance with the disposal of those amino acids.

Stable isotope tracer methodology is often used to measure amino acid kinetics (5). The basic principle is that the dilution measurement of the infused stable isotope amino acid makes it possible to calculate the endogenous substrate production and disposal, and when infusing stable isotopes of essential amino acids, PB can be estimated (6). Protein turnover in healthy individuals and during a variety of disease states, including critical illness, has predominantly been calculated using traditional methods such as primed-constant and continuous infusion of combinations of stable isotope amino acids such as leucine, phenylalanine, and tyrosine (7, 8). This approach requires intravenous infusion of stable amino acid tracers using a calibrated pump and accurate priming of the tracer pool to instantly obtain a tracer steady state, which is not always easy (4).

**Figure 1:**
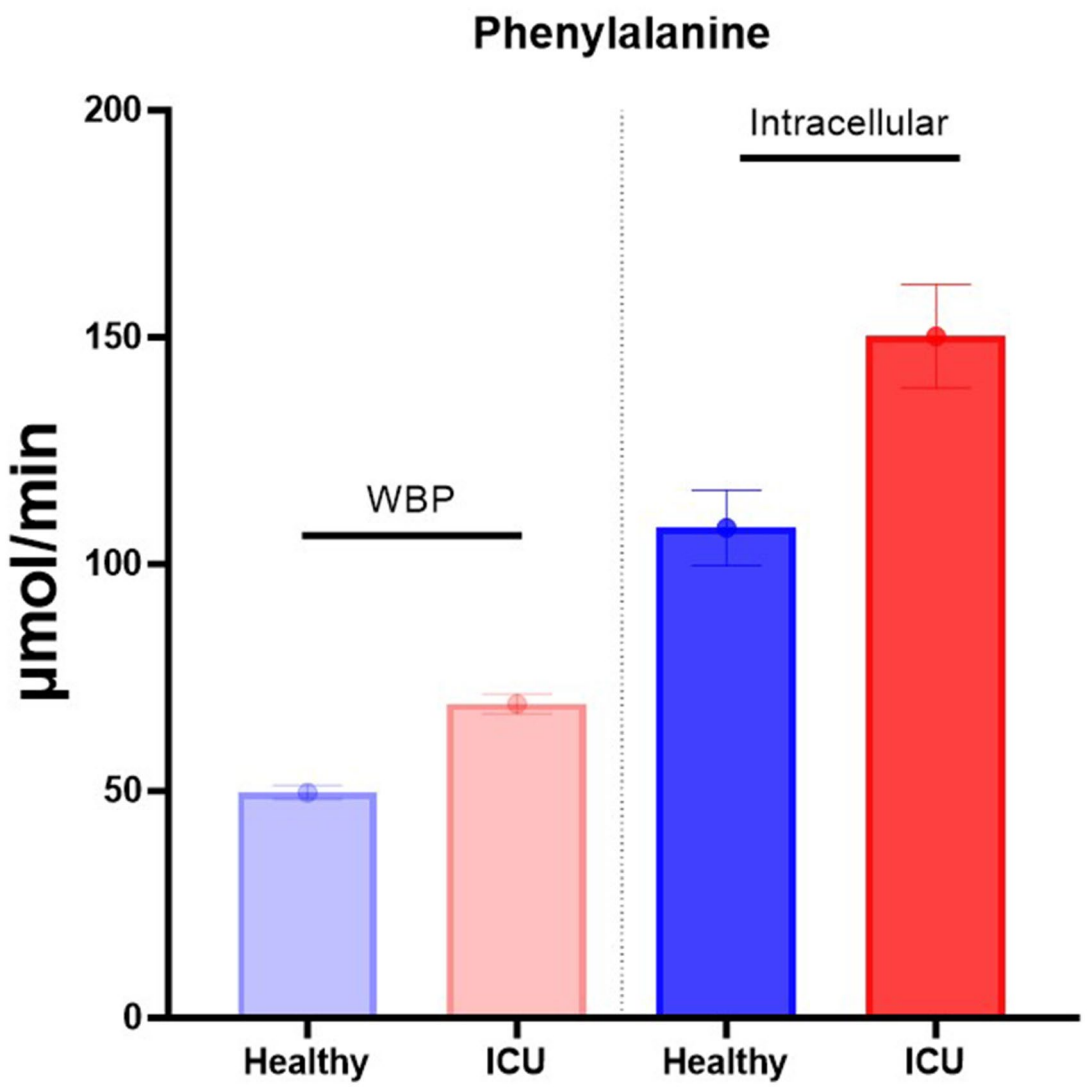
Intracellular phenylalanine production is much higher than WBP. Data are from Deutz et al. (4).

Recently, we reported a novel pulse tracer approach that enables the calculation of the intracellular production of amino acids (2–4, 9, 10). When using this approach, a pulse of stable isotopes is administered intravenously in a small volume and within 10 s. Measuring the decay of the isotope enrichments in plasma makes it possible to calculate simultaneously both the whole-body production (WBP) rate of these amino acids [is equal to the non-compartmental rate of appearance (Ra) as calculated by the primed-constant and continuous infusion model (7, 8)], and the intracellular production rate (3, 4) from the compartmental analysis. We have used this approach to compare the WBP and intracellular production of amino acids to better understand the balance between protein degradation rate and dietary protein intake (Figure 1).

## Protein turnover measurement in the post-absorptive state

As critically ill patients can have a wide range of changes in plasma amino acid concentrations (2, 11), correct priming of the pool to obtain instant tracer and tracer product steady state can be very difficult (12). To overcome this, we developed a pulse amino acid tracer approach (9), which does not need an infusion pump or knowledge of the pool sizes, and requires much smaller amounts of tracers than the traditional primed-constant and continuous infusion method. The pulse tracer approach is therefore an easy-to-use method for critically ill patients to study in depth their whole-body amino acid kinetics. Amino acid production can be assessed by calculating the area under the curve (AUC) of the tracer-tracee ratio decay (10) in the measured time period or by fitting the decay with a 2 exponential functions (3).

By measuring the phenylalanine production in healthy and critically ill patients (Figure 1), we can calculate the turnover of protein per gram protein/day/subject (Table 1 and Supplementary Figure S1) (4). As previously reported by others (13), ~300 grams of protein are broken down per day in a healthy individual in the postabsorptive state (4) as measured by the primed-constant and continuous stable isotope infusion protocol. If food only increases protein synthesis (see later), a dietary intake of approximately 75 grams of balanced protein would enhance protein synthesis to 375 grams, which is an ~25% increase.

**Table 1:** Recalculation of postabsorptive protein breakdown as grams of protein/day in humans.

	Healthy	ICU	ICU minus healthy
Non-compartmental PB (WBP)	292 [286, 305]	411 [398, 424]	115 [100, 131]
Non-compartmental net PB	44 [42, 47]	31 [29, 33]	−13 [−17, −10]
Intracellular PB	642 [592, 691]	892 [825, 960]	251 [167, 334]
Intracellular net PB	87 [82, 92]	60 [56, 65]	−26 [−33, −20]

Data are grams of protein/day [mean (95% CI)] protein breakdown (PB), obtained from recalculation of data, described previously (4). We used phenylalanine and tyrosine decay curve parameters to calculate the non-compartmental PB (comparable to the rate of appearance in plasma), intracellular production, and net protein breakdown, using the conversion of phenylalanine to tyrosine. We assumed that 4% of protein is phenylalanine.

The leucine/KIC approach (reciprocal model) (6) is often used to better estimate the total PB by assuming that the plasma KIC enrichment represents better the whole-body intracellular enrichment of leucine than the plasma leucine enrichment, as the conversion of leucine to KIC mainly takes place in muscle (14). PB measured with the KIC plasma enrichment is 1.3 times higher than when using the leucine plasma enrichment (15). In contrast, using the pulse approach, PB appears to be 2.6 times higher when calculated with the intracellular appearance of leucine than when calculated with Ra (4). Therefore, using plasma KIC enrichment to better estimate PB is not sufficient to correctly estimate intracellular PB. One of the reasons for this difference could be that it is based on the assumption that the conversion of leucine to KIC takes place in all organs at the same rate. Therefore, although this approach seems better than using plasma enrichment of leucine (6), it does not seem to correctly estimate intracellular PB.

As net PB depends on the careful measurement of oxidation when using the leucine/KIC approach, we and others used the combined phenylalanine/tyrosine approach, which only needs plasma enrichment measurements. The pulse approach with compartmental analysis and the phenylalanine/tyrosine approach, in our opinion, have the advantage of only requiring plasma enrichment measurements to calculate intracellular PB.

However, calculating PB from intracellular production (Table 1) leads to approximately 650 grams/day of PB in the postabsorptive state. In this case, a dietary intake of approximately 75 grams of protein/day would only lead to an 11% increase in protein synthesis. Consequently, if we calculate the net protein loss, which is the difference between PB and synthesis, a net loss of 87 grams of protein/day will take place in a healthy individual when there is no food intake. The net loss would be less if protein synthesis was lower in the absence of protein intake. However, during 12 h fasting (16), PB and oxidation of leucine are not increased, while after 3 days of fasting, protein oxidation is increased by 13% and PB by 30% (17). We therefore conclude that it is likely that the PB rate is not reduced by 24 h of fasting, and thus that our calculations of net loss are probably a good estimation.

In critically ill patients (Table 1), both non-compartmental PB and intracellular PB are increased by approximately 40% as compared to the healthy state. When using the intracellular PB measurement, protein degradation is approximately 900 grams/day and net protein loss is approximately 60 grams/day, which clearly shows that the turnover of protein is substantially increased in relation to the net loss. However, the net loss in critically ill patients is only 6.6% of total PB.

The calculations of net protein synthesis and breakdown are also affected by intracellular appearance. The calculation of phenylalanine hydroxylation as a proxy for oxidation is the enrichment ratio between plasma phenylalanine and the phenylalanine product tyrosine multiplied by the appearance of tyrosine. The conversion of phenylalanine to tyrosine occurs intracellularly, and thus the ratio estimates the correct ratio for both Ra and intracellular appearance calculations. However, the Ra of tyrosine underestimates the intracellular appearance of tyrosine, and therefore, the calculation of net protein synthesis/breakdown is higher than when the Ra of tyrosine is used.

One remarkable observation is that net PB in critically ill patients is not increased but decreased in the postabsorptive state (Table 1 (2–4)). We believe that we should try to interpret this observation physiologically. We hypothesize that reducing net protein loss in ICU patients could be a protective mechanism to reduce protein loss during disease. We also observed the same phenomenon in patients with chronic illnesses or at a higher age (18). Further research is needed to provide a more mechanistic explanation.

According to the calculations of the reduction of net PB in critically ill patients and thus of loss of lean mass, using the compartmental calculations (Table 2), critically ill patients will still lose approximately 400 grams of lean tissue/day (0.8%/day when total lean mass is approximately 50 kg). Others have found that loss of muscle mass, which is approximately 50% of total lean mass (19) in critically ill patients in the ICU is approximately 1%/day (20).

**Table 2:** Estimated net lean mass loss in grams of protein/day in humans when no food is provided.

Healthy	577 gram [544, 611]
Critically Ill	402 gram [373, 431]
ICU minus healthy	−175 gram [−220, −131]

Data are gram lean mass/day loss, calculated from intracellular net protein breakdown, assuming lean mass contains 15% protein.

So how can protein loss be attenuated in healthy subjects in the postabsorptive state? We calculated (Table 1) that net protein loss in healthy subjects is approximately 87 grams, indicating that at least 87 grams of dietary amino acids are needed for a healthy subject to become anabolic. When protein is ingested, other factors such as digestion play a leading role in reduced protein efficiency, which may partly explain the higher protein intake advised (21). Reduced digestibility of dietary proteins likely becomes even more important in critically ill patients.

What are the clinical implications of these observations (2–4)? If PB is much higher in critically ill patients than previously thought, the protein synthesis rate will also increase. The energy costs of protein synthesis are approximately 1.3 kcal/gram protein (22), suggesting that these energy costs of critically ill patients are approximately 900 × 1.3 kcal = 1,170 kcal, 65% of the total 1,800 kcal REE we measured in critically ill patients (2). In addition, we suggested that not so much the amount of protein intake needs to be increased in critically ill patients, but that particularly the upregulated PB needs to be reduced (4). Therefore, we hypothesize that critically ill patients need dietary components that can reduce PB. We recently showed a reduction in PB when providing HMB to critically ill patients (23). However, additional research is needed on whether certain dietary amino acids and/or proteins are also able to reduce PB, as we previously showed for arginine in the critically ill (24).

## Protein breakdown and synthesis during feeding

During feeding, there is an increase in amino acids released into the circulation, due to enhanced digestion and absorption of the meal-derived amino acids and from amino acids that become available from intracellular PB. The increased appearance of amino acids in the circulation and intracellularly will stimulate the disposal of amino acids (mainly for protein synthesis) and result in an increased intracellular concentration that could reduce PB. Several studies, including our own, have observed a reduced endogenous PB when using the primed-constant and continuous tracer infusion model (7, 8).

One important complicating factor could be the splanchnic extraction of amino acids that could affect the dilution of plasma enrichment. Endogenous PB is the rate of appearance (Ra), corrected for the amount of tracee entering the whole-body pool from nutrition. So we need to establish how much of the meal-derived amino acids are absorbed in the gut. If we assume that absorption is 100% for free dietary amino acids, the amount of nutrition entering the body pool in the mucosa cell needs to be subtracted from the Ra (calculated from the primed-continuous or pulse approach) or the intracellular appearance (from the pulse approach) to estimate endogenous PB.

So how does splanchnic extraction of meal-derived amino acids (e.g., phenylalanine) play a role in calculating endogenous PB? The basic assumption is that when using the Ra, a correction needs to be made for the Ra with the rate of meal-derived phenylalanine, appearing in the hepatic vein (thus post-splanchnic). The misunderstanding with this approach could be that the Ra only represents the PB of non-splanchnic organs and that subtracting the post-splanchnic appearance corrects the Ra on a whole-body level. In actuality, the Ra includes PB in all organs, and thus the absorbed meal-derived amino acids into the mucosa cell should be subtracted to calculate endogenous PB.

So what is the role of the splanchnic extraction measurement with the continuous stable isotope tracer infusion approach during feeding? In our opinion, this calculation is not needed at all. Only the estimation of how many amino acids from food are absorbed should be sufficient (6) to calculate endogenous PB. Using the pulse tracer approach, the same arguments will hold. There is no need to estimate splanchnic extraction to estimate endogenous PB. Therefore, the calculation that was used for many years, Ra = protein synthesis + oxidation = protein breakdown + intake from food remains valid (7, 25, 26). Using the pulse approach, Ra is replaced by the intracellular appearance.

However, it remained unclear whether the proteins in the meal were indeed able to reduce PB. We recently performed a pilot study on 11 human subjects to examine whether intracellular PB (using compartmental analysis) can also be measured in the prandial state (27, 28). For that purpose, we developed a protocol in which nutrition was provided every 20 min as sips containing a mixture of free amino acids, representing the composition of whey protein. Nutrition needs to be given as sips to obtain a steady state influx of dietary amino acids, and we previously observed that using a sip protocol gives comparable information on net protein synthesis and other measures, with some caveats (8). After the steady state was obtained, as verified by adding stable isotopes of amino acids to the sips and measuring the plasma enrichment and concentration of these amino acids, we administered the pulse of stable isotopes as previously conducted in the postabsorptive condition.

**Figure 2:**
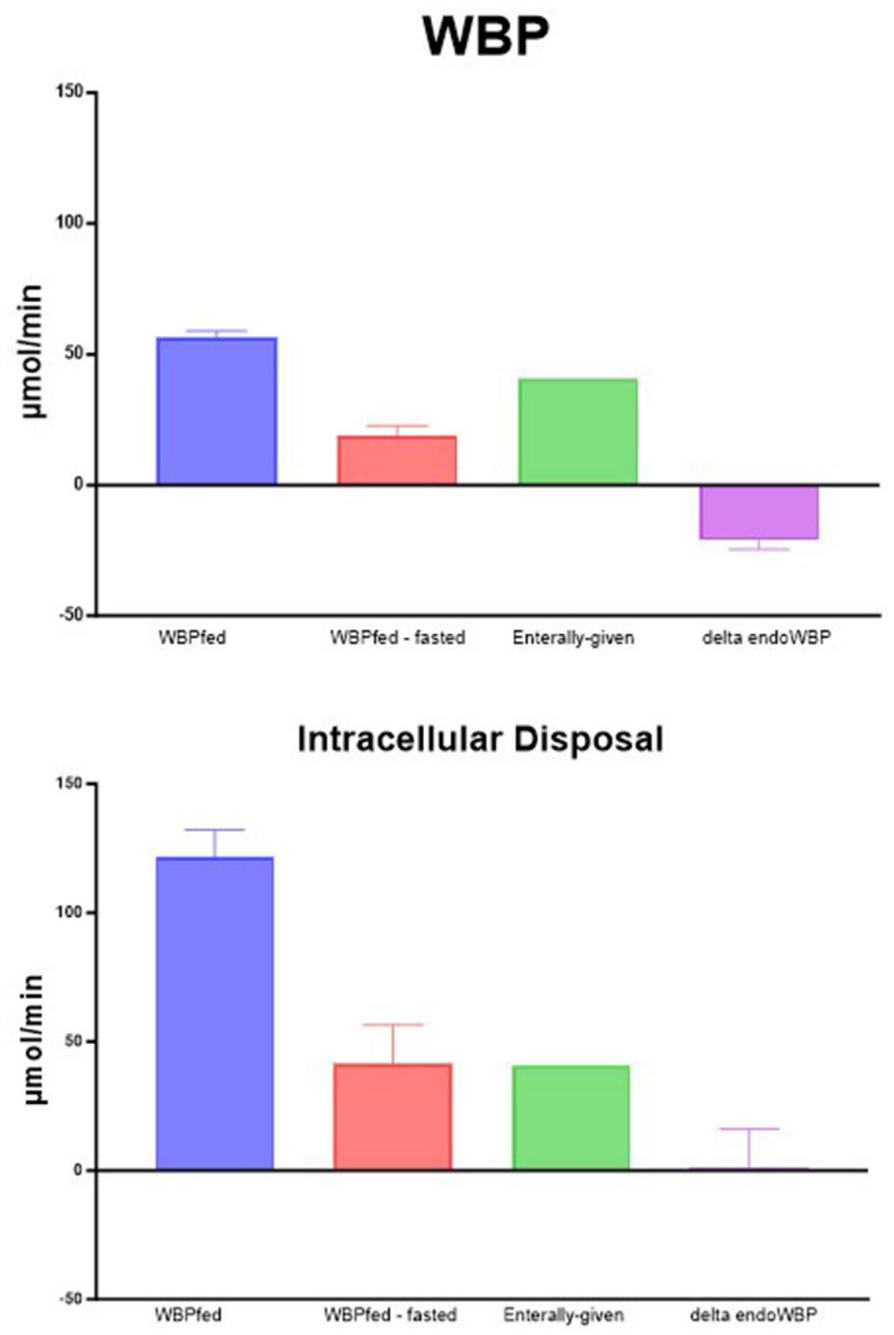
Phenylalanine production. WBPfed is the non-compartmental production during feeding. WBPfed—fasted is when the WBP in the post-absorptive condition is subtracted from the WBP during feeding. Enteral-given is the amount of phenylalanine given with the food. Delta EndoWBP is enterally given and subtracted from WBPfed—fasted, representing the change in the endogenous protein breakdown.

We subsequently compared the turnover of phenylalanine (as a measure of PB) obtained by non-compartmental and intracellular (compartmental) analyses in the prandial state (Figure 2, upper panel). Non-compartmental protein breakdown (WBP), measured during feeding (WBPfed), was approximately 50 μmol/min using this approach. When subtracting the WBP when no food was given (WBPfed—fasted), the difference in WBP was approximately 20 μmol/min. As the amount of phenylalanine given enterally was greater than the increase in WBPfed—fasted, the difference became negative (delta endoWBP). This means that there is a reduction in endogenous protein breakdown (endoWBP) when calculating the effect of feeding. A consistent reduction of PB during feeding has previously been observed by us (7, 8) and others (29–31) when WBP was measured using the primed-constant and continuous infusion models.

However, as indicated above, the non-compartmental protein breakdown calculation (WBP) underestimates the true intracellular PB, suggesting that our calculation of the intracellular PB provides a better reflection of the true PB. When the same calculations were performed using the intracellular PB approach (Figure 2, lower panel), the amount of phenylalanine given enterally as sips was very well matched with the difference between the intracellular appearance fed and fasted. Therefore, no reduction in PB was observed anymore. Therefore, we believe that feeding does not reduce endogenous PB and that these findings in the past might have likely been caused by the stable isotope tracer model used.

## Perspectives and limitations

Our approach of combining sip feeding with the isotope pulse method can be used to measure the intracellular appearance of amino acids from any food protein. The intracellular appearance depends on how many amino acids are left after digestion and absorption of the food protein, the appearance of the plasma pool, and other factors that could have reduced the intracellular appearance and availability. Our pilot study shows that when using a mixture of free dietary amino acids and assuming that the digestion and absorption of free amino acids are not limited, the intracellular appearance of amino acids matches the amount consumed. However, a limitation of our observation is that we still do not know the exact digestion and absorption rates of amino acids.

Furthermore, when consuming a meal with a certain amount of protein, the biological value of that protein in principle can be calculated with our new approach, but this needs more validation studies. The same is true for complex meals with different types of proteins. Protein synthesis stimulatory effects of a meal seem to be an important factor in calculating the exact protein requirements and needs.

Therefore, we propose to check our approach in critically ill patients during feeding to establish which factors affect the anabolic capabilities of certain dietary amino acid mixtures or proteins as it might guide nutritional approaches in the critically ill.

## Conclusion

We have provided a new view on protein metabolism in the postabsorptive and fed state when using the pulse stable isotope tracer approach. We have concluded that the estimation of protein turnover with the primed-constant and continuous infusion protocol is too low and healthy human PB is more in the range of 600 grams of protein/day.

We also presented new data that show that during feeding, endogenous PB is likely not reduced by food, but that feeding only stimulates protein synthesis.

## Author contributions

ND: Writing – original draft, Writing – review & editing. ME: Writing – original draft, Writing – review & editing.

## Supplementary material

SUPPLEMENTARY FIGURE S1 Graphic representation of the data as shown in Table 1.
