# Supplementary figures and images for: Compartmental analysis: a new approach to estimate protein breakdown and meal response in health and critical illness
Source: Front Nutr. 2024 May 9;11:1388969. doi: 10.3389/fnut.2024.1388969 (PMC11111962; doi:10.3389/fnut.2024.1388969)

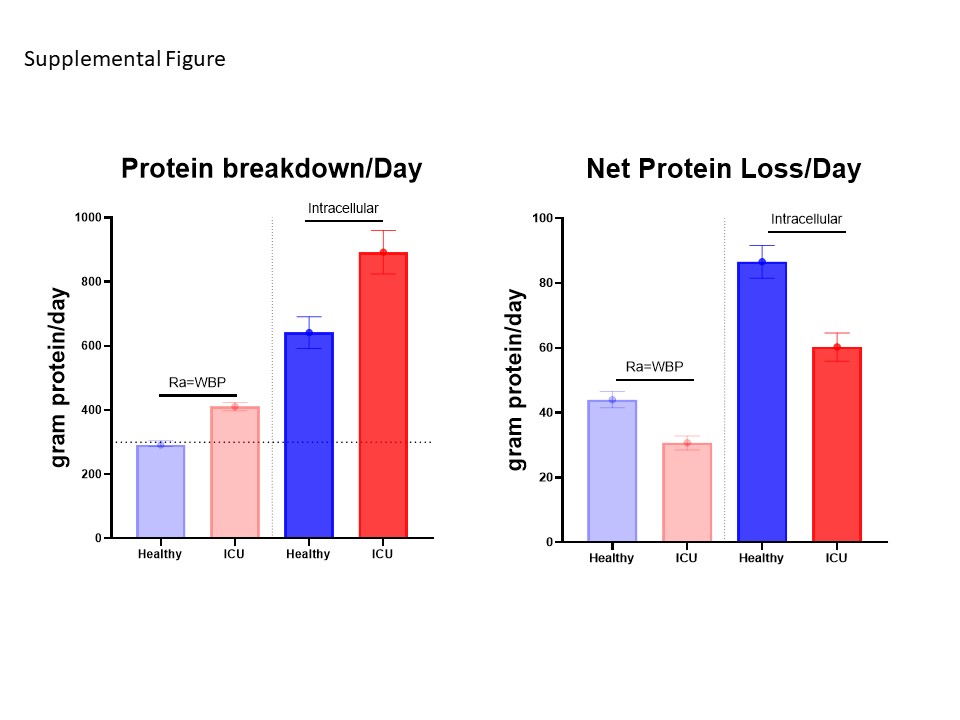

Supplement: SUPPLEMENTARY FIGURE S1 — Graphic representation of the data as shown in Table 1. [file Image_1.JPEG]
